# Decreased Serum Levels of SIRT1 and SIRT3 Correlate with Severity of Skin and Lung Fibrosis and Peripheral Microvasculopathy in Systemic Sclerosis

**DOI:** 10.3390/jcm11051362

**Published:** 2022-03-01

**Authors:** Mirko Manetti, Irene Rosa, Bianca Saveria Fioretto, Marco Matucci-Cerinic, Eloisa Romano

**Affiliations:** 1 Section of Anatomy and Histology, Department of Experimental and Clinical Medicine, University of Florence, 50134 Florence, Italy; irene.rosa@unifi.it (I.R.); biancasaveria.fioretto@unifi.it (B.S.F.); 2 Division of Rheumatology, Department of Experimental and Clinical Medicine, University of Florence, 50134 Florence, Italy; marco.matuccicerinic@unifi.it (M.M.-C.); eloisa.romano@unifi.it (E.R.); 3 Unit of Immunology, Rheumatology, Allergy and Rare Diseases (UnIRAR), IRCCS San Raffaele Hospital, 20132 Milan, Italy

**Keywords:** systemic sclerosis, scleroderma, sirtuins, SIRT1, SIRT3, skin fibrosis, pulmonary fibrosis, peripheral microvascular damage, enzyme-linked immunosorbent assay

## Abstract

Systemic sclerosis (SSc, scleroderma) is a severe autoimmune connective tissue disease characterized by widespread peripheral microvasculopathy, and progressive cutaneous and visceral fibrosis, leading to significant organ dysfunction. Sirtuins (SIRTs) are a family of NAD-dependent protein deacetylases with pleiotropic effects on a variety of biological processes, including metabolism, cell survival, and aging. In the last decades, increasing studies have explored the contribution of SIRTs to the pathogenesis of SSc, highlighting a significant antifibrotic effect of both SIRT1 and SIRT3. On these bases, the aim of this study was to measure circulating SIRT1 and SIRT3 levels by enzyme-linked immune-sorbent assay in a well-characterized cohort of SSc patients (*n* = 80) and healthy controls (*n* = 71), focusing on their possible association with disease clinical features, and their potential as biomarkers reflecting SSc activity and severity. Significantly decreased serum levels of both SIRT1 and SIRT3 were found in SSc patients compared to controls. In SSc, the reduction in circulating SIRT1 and SIRT3 associated with a greater extent of cutaneous fibrosis, presence of interstitial lung disease, and worse pulmonary function. Serum SIRT1 and SIRT3 decrease also correlated with the severity of nailfold microvascular damage, with SIRT3 levels being additionally related to the occurrence of digital ulcers. The levels of these two proteins showed a direct correlation with one another in the circulation of SSc patients. Of the two SIRTs, serum SIRT3 was found to better reflect disease activity and severity in a logistic regression analysis model. Our findings suggest that serum SIRT1 and SIRT3 may represent novel potential biomarkers of increased risk for a more severe, life-threatening SSc disease course.

## 1. Introduction

Systemic sclerosis (SSc, scleroderma) is a severe autoimmune disease characterized by widespread microvasculopathy, clinically manifesting with Raynaud’s phenomenon, refractory ischemic digital ulcers (DUs), pulmonary artery hypertension (PAH), and renal crisis, as well as by cutaneous and visceral fibrosis, finally leading to significant organ dysfunction [1,2,3,4]. Such a highly heterogeneous spectrum of pathological manifestations associates with a widely variable prognosis, with lung fibrosis that clinically manifests as interstitial lung disease (ILD) representing the principal cause of death [5,6,7]. According to the extent of skin fibrosis, SSc can be classified into two main clinical subsets, namely limited cutaneous SSc (lcSSc) and diffuse cutaneous SSc (dcSSc). In both, the fibrotic process starts at the distal ends of the extremities, and progresses centripetally, but if in lcSSc patients, skin fibrosis remains confined to areas distal to elbows or knees, with or without involvement of the face, in dcSSc patients, cutaneous fibrosis may also affect the arms, legs, and trunk [8]. In addition, dcSSc patients are characterized by a rapid progression of skin involvement with early internal organ disease, whereas in patients with the lcSSc subset, the course of the disease is protracted over time [8]. Concerning SSc histopathology, skin and internal organ fibrosis is driven by profibrotic myofibroblasts, i.e., α-smooth muscle actin expressing cells whose persistent activation results in excessive synthesis and accumulation of extracellular matrix components, such as collagen and fibronectin, with consequent disrupted physiological architecture of the affected tissues [9,10].

Sirtuins (SIRTs) are a family of NAD-dependent protein deacetylases with pleiotropic effects on a variety of key biological processes, including metabolism, cell survival, and aging [11,12]. Of the seven conserved SIRT isotypes (SIRT1–SIRT7), SIRT1, 6, and 7 are principally nuclear, whereas others are mainly located in the cytoplasm (SIRT2) or within mitochondria (SIRT3, 4, and 5) [11]. Accumulating literature indicates that SIRTs may regulate important fibrosis-mediating molecules and pathways, such as peroxisome proliferator-activated receptor γ, nuclear factor-κB, Notch and transforming growth factor β (TGFβ), with the majority of the studies reporting the existence of a link between decreased SIRT levels and the development of tissue fibrosis, and a protective and antifibrotic role of SIRT expression restoration [13]. In particular, the activation of SIRTs in vitro or in vivo has been shown to exert antifibrotic effects in experimental models of cardiac, renal, and pulmonary fibrosis [14,15,16,17,18].

In the last decades, increasing studies have explored the contribution of SIRTs to the pathogenesis of SSc, establishing a connection between a decrease in SIRT expression and disease-related fibrosis [13,19,20,21]. In this context, SIRT1 expression levels were found to be significantly reduced in the skin and dermal fibroblasts of SSc patients, as well as in the lesional skin from mice with bleomycin-induced scleroderma, with SIRT1 activation leading to a mitigation of fibrosis both in vitro and in vivo [13,19]. A similar decrease in SIRT1 levels was also detected in lung fibroblasts explanted from SSc patients with ILD, as well as in lung tissue from bleomycin-treated mice [22]. In addition, SIRT1 mRNA levels were found to be lower in circulating mononuclear cells of SSc patients with ILD in respect to those without pulmonary involvement, a downregulation that was found to be preferentially associated with the dcSSc subset and anti-topoisomerase I antibody (ATA) positivity [7]. Interestingly, activation of SIRT1 with resveratrol was reported to ameliorate both lung and dermal fibrosis in bleomycin-induced scleroderma mice through the inhibition of TGFβ signaling, or by targeting the mTOR pathway, respectively [7,23]. Another SIRT that has been recently investigated in SSc due to its supposed antifibrotic effects is SIRT3. The expression of SIRT3 was found to be impaired both in SSc skin biopsies and dermal fibroblasts and, similarly, the development of skin fibrosis was paralleled by a significant downregulation of SIRT3 within the lesional dermis of bleomycin-treated mice [21]. In the same study, the authors demonstrated that incubation of SSc fibroblasts with hexafluoro, a synthetic honokiol derivative able to induce SIRT3 expression and activity, attenuated the activated phenotype of such cells [21]. Moreover, treatment with hexafluoro mitigated both lung and skin fibrosis in the bleomycin-induced scleroderma mouse model [21]. A significant decrease in SIRT3 levels were finally reported in lung tissue from SSc patients, as well as in two murine models of pulmonary fibrosis [24]. Finally, SIRT3-deficient mice showed an increased susceptibility to pulmonary fibrosis that was related to enhanced canonical TGFβ signaling [24].

On the bases of this scientific background, the objective of the present study was to measure, for the first time, circulating SIRT1 and SIRT3 levels by enzyme-linked immune-sorbent assay (ELISA) in a well-characterized cohort of SSc patients, focusing on their possible association with disease clinical subsets and features, and their potential as biomarkers reflecting SSc activity and severity.

## 2. Materials and Methods

### 2.1. Patients, Controls, and Serum Samples

Serum samples were obtained from 80 patients fulfilling the ACR/EULAR 2013 classification criteria for SSc [25] (74 women and 6 men; mean ± SD age 58.3 ± 13.6 years), and recruited from the Division of Rheumatology, University of Florence. Patients with symptoms overlapping with those of other autoimmune, rheumatic, and/or connective tissue disorders were not enrolled in the study. SSc patients were not taking corticosteroids, methotrexate, cyclophosphamide, ACE inhibitors, D-penicillamine, iloprost, or other immunosuppressive treatment and disease-modifying drugs. Seventy-one age- and sex-matched healthy individuals (67 women, 4 men; mean ± SD age 59.7 ± 12.8 years) were used as controls. Primary Raynaud’s phenomenon was considered as an exclusion criterion for healthy controls. Fresh peripheral blood samples from patients and controls were drawn, allowed to clot for 30 min, and centrifuged at 1500× *g* for 15 min, followed by serum collection and storage in aliquots at −80 °C until used. The study was conducted in accordance with the Declaration of Helsinki, and approved by the local Institutional Review Board. All subjects provided written informed consent.

### 2.2. Clinical Assessment

The totality of patients was classified as having lcSSc (*n* = 47) or dcSSc (*n* = 33) according to the criteria of LeRoy et al. [26], and clinically assessed as recommended elsewhere [27]. Anticentromere antibodies (ACAs) were evaluated by their distinctive indirect immunofluorescence pattern on HEp-2 cells, whereas ATAs were detected by immunoblot analysis. The extent of cutaneous fibrosis was determined by means of the modified Rodnan skin thickness score (mRSS) through palpation of the skin in 17 body areas (i.e., fingers, hands, forearms, arms, feet, legs, thighs, face, chest, and abdomen) using a 0–3 scale (i.e., 0 = normal, 1 = mild thickness, 2 = moderate thickness, and 3 = severe thickness), with total score ranging from 0 (no thickening) to 51 (severe thickening in all 17 body areas) [28]. The presence of typical ILD features, such as bibasilar interstitial fibrosis, was evaluated in all SSc patients through a high-resolution computed tomography (HRCT) scan of the chest. Moreover, forced vital capacity (FVC) and diffusing capacity of the lungs for carbon monoxide (DLCO) pulmonary function tests were performed as measures of lung fibrosis severity [29]. Levels of FVC and DLCO ≤ 70% of the predicted normal values were considered abnormal. Patients with SSc who were smokers or presented other respiratory disorders possibly affecting FVC or DLCO were excluded from the study. Doppler echocardiography was used to screen for PAH, and, once identified, it was then confirmed with right heart catheterization. None of the patients had scleroderma renal crisis. All SSc patients reported the occurrence of Raynaud’s phenomenon. The presence of ischemic DUs on the fingertips and other finger areas was recorded at the time of peripheral blood drawing. Finally, microvascular abnormalities on all 10 fingers were analyzed by nailfold videocapillaroscopy (NVC). After allowing patients to adapt to room temperature (20–22 °C) for at least 15 min before the examination, their nailfolds were analyzed for the presence of pericapillary edema, microhemorrhages, enlarged and giant capillaries, ramified or bushy capillaries, disorganization of the microvascular distribution, and loss of capillaries. Three different NVC patterns were identified: (i) “early” NVC pattern, characterized by few enlarged/giant capillaries and capillary microhemorrhages, no evident capillary loss, and a relatively well-preserved capillary distribution; (ii) “active” NVC pattern, with giant capillaries and capillary microhemorrhages, absence or presence of few ramified capillaries, moderate capillary loss, and mild disorganization of the capillary architecture; and (iii) “late” NVC pattern, featuring irregular capillary enlargement, absence or presence of few giant capillaries, absence of microhemorrhages, frequent ramified/bushy capillaries, severe loss of capillaries with large avascular areas, and disorganization of the normal capillary array [30]. A summary of the main characteristics of patients with SSc is shown in Table 1.

### 2.3. ELISA for Serum SIRT1

Serum levels of SIRT1 were measured with a commercial quantitative colorimetric sandwich ELISA kit featuring an antigen-affinity purified antihuman SIRT1 capture antibody precoated 96-well plate and a biotin-conjugated antibody as detection antibody (catalog no. EKF57715; Biomatik, Wilmington, DE, USA), according to the manufacturer’s protocol. Standards and serum samples were incubated for 90 min at 37 °C, and, after two washes, the biotin-labeled antibody was added to the plate and incubated for 60 min at 37 °C. The reaction was developed with streptavidin-horseradish peroxidase conjugate and tetramethylbenzidine, and then stopped by applying 2 M H_2_SO_4_. Optical density was measured through microtiter plate reader at 450 nm. SIRT1 serum levels were read off from a standard curve following the manufacturer’s instructions. The detection range and the sensitivity of the assay were 0.313–20 ng/mL and 0.188 ng/mL, respectively. Each serum sample was tested in duplicate. The whole serum sample series were assayed at the same time using ELISA kits of the same batch.

### 2.4. ELISA for Serum SIRT3

Serum levels of SIRT3 were measured with a commercial ELISA kit (catalog no. EKN48463; Biomatik, Kitchener, ON, Canada) following the manufacturer’s instructions. In particular, standards or serum samples were added to a microplate precoated with an antibody specific to SIRT3, and allowed to incubate for 60 min at 37 °C. A biotin-conjugated antibody was used as detection antibody (60 min at 37 °C incubation). The reaction, developed by adding avidin-horseradish peroxidase conjugate (30 min at 37 °C incubation) and tetramethylbenzidine (20 min at 37 °C incubation), was terminated by applying 2 M H_2_SO_4_. Color change was measured spectrophotometrically at 450 nm. SIRT3 concentrations were finally determined by comparing the optical density of each sample to the standard curve. The detection range and the sensitivity of the assay were 0.156–10 ng/mL and 0.061 ng/mL, respectively. Each serum sample was analyzed in duplicate. The whole serum sample series were assayed at the same time using ELISA kits of the same batch.

### 2.5. Statistical Analysis

Statistical analysis was carried out with the SPSS software for Windows V27.0 (SPSS, Chicago, IL, USA). Descriptive statistics were expressed as mean ± SD or median and interquartile range (IQR) for continuous variables, and as number and percentage for categorical variables. The accuracy of serum SIRT1 and SIRT3 levels for the diagnosis of SSc disease was estimated by performing receiver operator characteristics (ROC) curve analysis. A non-parametric Mann–Whitney U test for independent samples was performed to analyze differences in serum levels of SIRT1 or SIRT3 between two groups. The Spearman ρ correlation coefficient was calculated to study the relationship between two continuous variables. Since Mann–Whitney U test analyses revealed that serum levels of both SIRTs were significantly decreased in patients with the dcSSc subset, patients with ILD, and patients with an “active/late” NVC pattern, we performed multiple logistic regression analysis including SIRT1 and SIRT3 as independent variables and a single dependent variable each time (i.e., cutaneous subset, presence of ILD, and NVC pattern). Odds ratios (ORs) with 95% confidence intervals (95% CIs) were determined. All *p* values are two-tailed, and the statistical significance level was set at *p* < 0.05.

## 3. Results

### 3.1. Serum Levels of SIRT1 Are Decreased in SSc Patients

**Figure 1:**
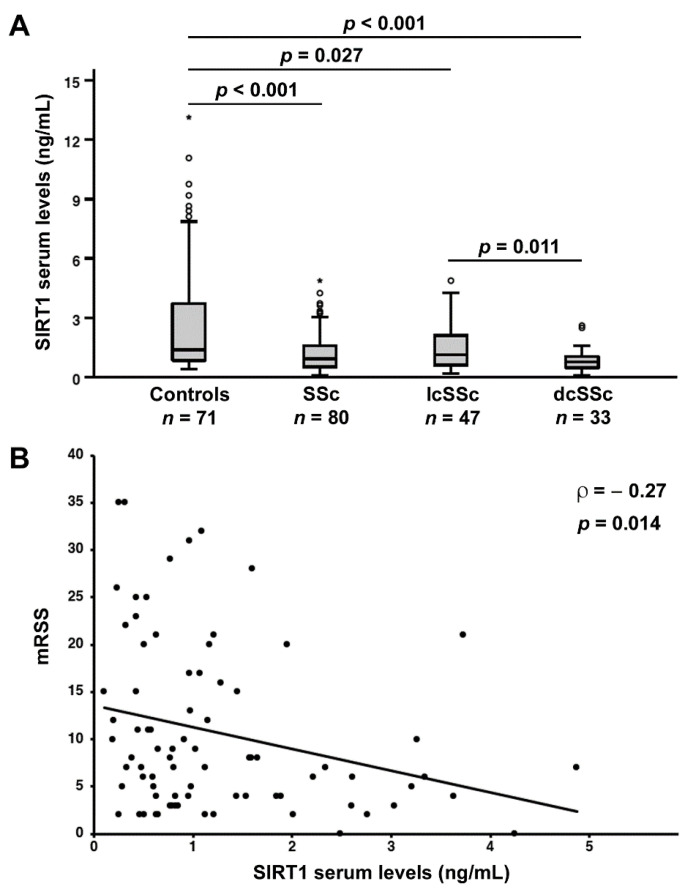
Serum levels of sirtuin 1 (SIRT1) are decreased, and correlate with the extent of skin fibrosis in systemic sclerosis (SSc) patients. (**A**) Serum SIRT1 levels in healthy controls, SSc patients and limited cutaneous SSc (lcSSc), and diffuse cutaneous SSc (dcSSc) subsets. Data are presented as box plots. Each box denotes the 25th to 75th percentiles. Lines outside the boxes are the 10th and 90th percentiles. Lines inside the boxes denote the median, circles denote the outliers, and asterisks denote the extreme values. (**B**) Correlation of SIRT1 levels with modified Rodnan skin thickness score (mRSS) in SSc patients. Data are displayed as scatterplot, where each dot represents a patient. Correlation coefficient (ρ) and *p* values are indicated.

Circulating levels of SIRT1 were significantly decreased in SSc patients (median 0.94 ng/mL, IQR 0.53–1.59 ng/mL) compared to healthy controls (median 1.38 ng/mL, IQR 0.84–3.72 ng/mL; *p* < 0.001; Figure 1A). When we performed ROC curve analysis, we found that the area under the curve (AUC) of SIRT1 was 0.68 (95% CI 0.60–0.77), indicating a low diagnostic accuracy.

Next, we examined the possible association of circulating SIRT1 levels with different SSc clinical phenotypes. As far as SSc cutaneous subsets are concerned, serum SIRT1 levels in patients with lcSSc (median 1.15 ng/mL, IQR 0.60–2.21 ng/mL) and dcSSc (median 0.77 ng/mL, IQR 0.47–1.06 ng/mL) were both significantly lower than in healthy individuals (*p* = 0.027 and *p* < 0.001, respectively; Figure 1A). Patients with dcSSc had lower SIRT1 levels compared with lcSSc patients (*p* = 0.011; Figure 1A). No significant differences in SIRT1 levels were detected when patients were stratified according to autoantibody positivity. In fact, SIRT1 levels were similar in ACA+ patients (median 0.78 ng/mL, IQR 0.44–1.84 ng/mL) and ACA– patients (median 0.97 ng/mL, IQR 0.63–1.59 ng/mL; *p* > 0.05), as well as in ATA+ patients (median 0.96 ng/mL, IQR 0.49–1.55 ng/mL) and ATA– patients (median 0.84 ng/mL, IQR 0.53–1.85 ng/mL; *p* > 0.05).

### 3.2. Association of Circulating SIRT1 Levels with the Severity of Skin and Lung Fibrosis

When investigating the possible correlation of SIRT1 levels with the extent of skin fibrosis measured by mRSS, we found that serum SIRT1 inversely correlated with mRSS (ρ = −0.27, *p* = 0.014; Figure 1B).

**Figure 2:**
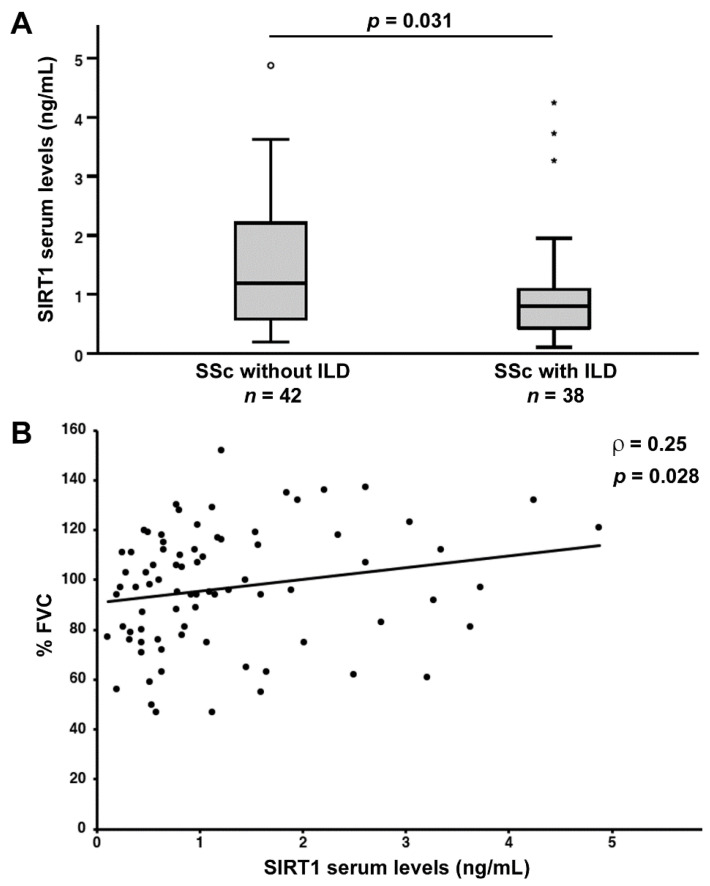
(**A**) Decreased serum levels of sirtuin 1 (SIRT1) in systemic sclerosis (SSc) patients with features of interstitial lung disease (ILD) on high-resolution computed tomography scan of the chest. Data are presented as box plots. Each box denotes the 25th to 75th percentiles. Lines outside the boxes are the 10th and 90th percentiles. Lines inside the boxes denote the median, circles denote the outliers, and asterisks denote the extreme values. (**B**) Correlation of serum SIRT1 levels with pulmonary function measured as percentage forced vital capacity (%FVC) in patients with SSc. Data are displayed as scatterplot, where each dot represents a patient. Correlation coefficient (ρ) and *p* values are indicated.

Serum SIRT1 levels were reduced in SSc patients diagnosed with ILD on HRCT scans of the chest (median 0.80 ng/mL, IQR 0.42–1.10 ng/mL) compared to patients without ILD (median 1.19 ng/mL, IQR 0.57–2.24 ng/mL; *p* = 0.031; Figure 2A). Moreover, serum levels of SIRT1 measured in SSc patients positively correlated with %FVC (ρ = 0.25, *p* = 0.028; Figure 2B). Conversely, no significant correlation was detected between SIRT1 and %DLCO (data not shown).

### 3.3. Association of Serum SIRT1 Levels with the Severity of Peripheral Microvascular Damage

**Figure 3:**
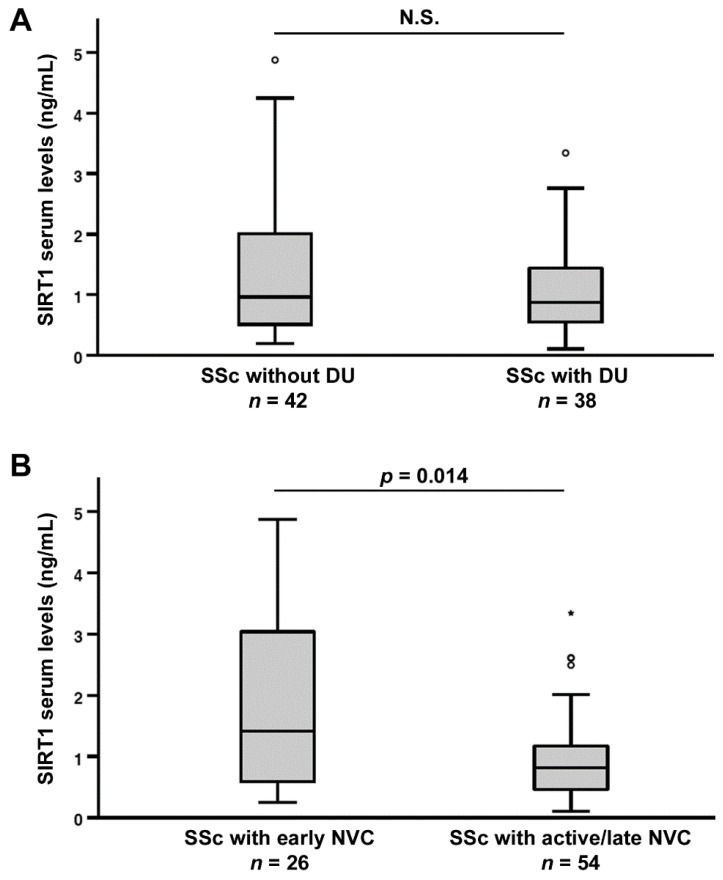
Association of serum sirtuin 1 (SIRT1) levels with the severity of peripheral microvascular damage in systemic sclerosis (SSc) patients. (**A**) SIRT1 levels in SSc patients with and without ischemic digital ulcers (DUs). (**B**) SIRT1 levels in SSc patients stratified according to “early” and “active/late” nailfold videocapillaroscopy (NVC) patterns. Data are presented as box plots. Each box denotes the 25th to 75th percentiles. Lines outside the boxes are the 10th and 90th percentiles. Lines inside the boxes denote the median, circles denote the outliers, and asterisks denote the extreme values; *p* values are indicated. N.S., not significant.

As a measure of peripheral microvascular involvement, we next investigated the possible correlation between circulating SIRT1, and both the presence of ischemic DUs and the NVC pattern. No significant difference in SIRT1 levels was detected when SSc patients were stratified according to the presence of DUs (Figure 3A), whereas SIRT1 was significantly higher in SSc patients with “early” NVC (median 1.41 ng/mL, IQR 0.59–3.08 ng/mL) than in those with “active/late” NVC (median 0.82 ng/mL, IQR 0.46–1.18 ng/mL) patterns (*p* = 0.014; Figure 3B).

No significant difference in SIRT1 levels was detected between SSc patients with PAH (median 0.97 ng/mL, IQR 0.49–1.87 ng/mL) and those without PAH (median 0.93 ng/mL, IQR 0.52–1.58 ng/mL; *p* > 0.05).

### 3.4. Circulating SIRT3 Levels Are Decreased in SSc Patients

**Figure 4:**
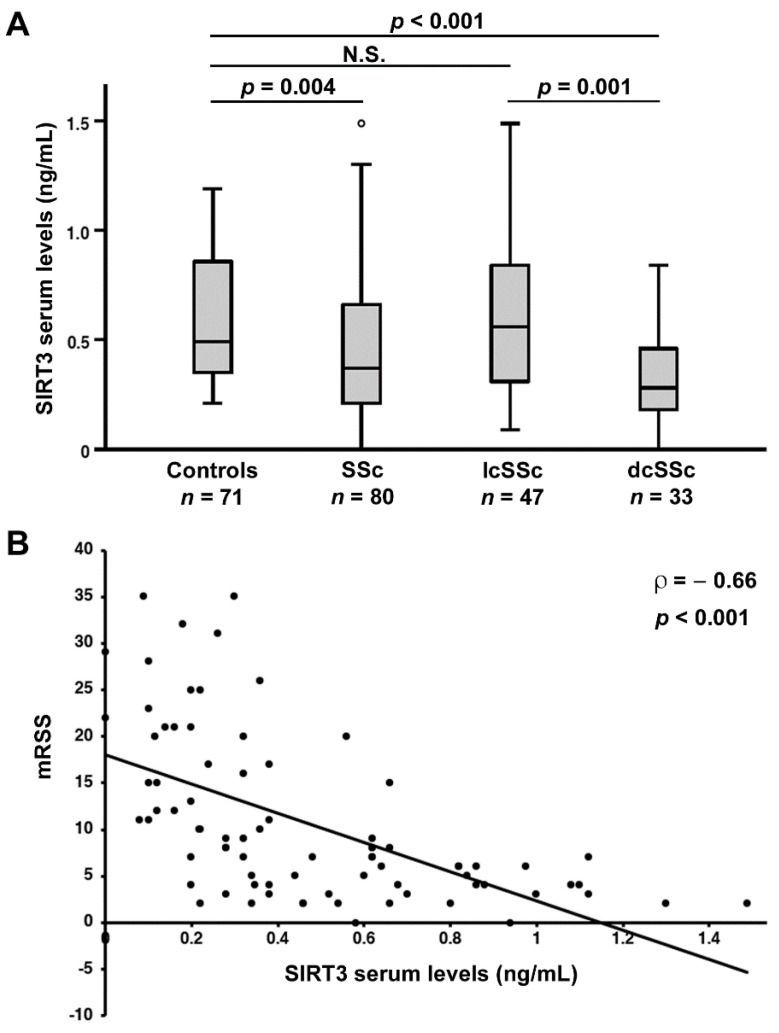
Serum levels of sirtuin 3 (SIRT3) are decreased, and correlate with the extent of skin fibrosis in systemic sclerosis (SSc) patients. (**A**) Serum SIRT3 levels in healthy controls, SSc patients and limited cutaneous SSc (lcSSc), and diffuse cutaneous SSc (dcSSc) subsets. Data are presented as box plots. Each box denotes the 25th to 75th percentiles. Lines outside the boxes are the 10th and 90th percentiles. Lines inside the boxes denote the median, circles denote the outliers, and asterisks denote the extreme values. (**B**) Correlation of SIRT3 levels with modified Rodnan skin thickness score (mRSS) in SSc patients. Data are displayed as scatterplot, where each dot represents a patient. Correlation coefficient (ρ) and *p* values are indicated. N.S., not significant.

SIRT3 levels were found to be significantly lower in patients with SSc (median 0.37 ng/mL, IQR 0.20–0.66 ng/mL) compared to controls (median 0.49 ng/mL, IQR 0.35–0.88 ng/mL; *p* = 0.004; Figure 4A). When we performed ROC curve analysis, we found that the AUC of SIRT3 was 0.64 (95% CI 0.55–0.72), indicating a low diagnostic accuracy.

We then explored the possible association of circulating SIRT3 levels with different SSc clinical phenotypes. When stratifying patients according to the SSc cutaneous subsets, we found that SIRT3 levels were significantly lower in dcSSc (median 0.28 ng/mL, IQR 0.17–0.47 ng/mL), but not in lcSSc patients (median 0.56 ng/mL, IQR 0.30–0.86 ng/mL) compared with controls (*p* < 0.001; Figure 4A). In addition, patients with dcSSc had lower SIRT3 levels compared to those with lcSSc (*p* = 0.001; Figure 4A). As reported for SIRT1, no significant differences in SIRT3 serum levels were found when patients were stratified according to autoantibody positivity. Indeed, SIRT3 levels were similar in ACA+ patients (median 0.46 ng/mL, IQR 0.28–0.82 ng/mL) and ACA– patients (median 0.32 ng/mL, IQR 0.20–0.62 ng/mL; *p* > 0.05), as well as in ATA+ patients (median 0.33 ng/mL, IQR 0.23–0.62 ng/mL) and ATA– patients (median 0.47 ng/mL, IQR 0.20–0.68 ng/mL; *p* > 0.05).

### 3.5. Association of Circulating SIRT3 Levels with the Severity of Skin and Lung Fibrosis

The possible correlation between SIRT3 levels and mRSS as a measure of the extent of SSc-related skin fibrosis was also evaluated. In particular, SIRT3 was found to negatively correlate with mRSS (ρ = −0.66, *p* < 0.001; Figure 4B).

**Figure 5:**
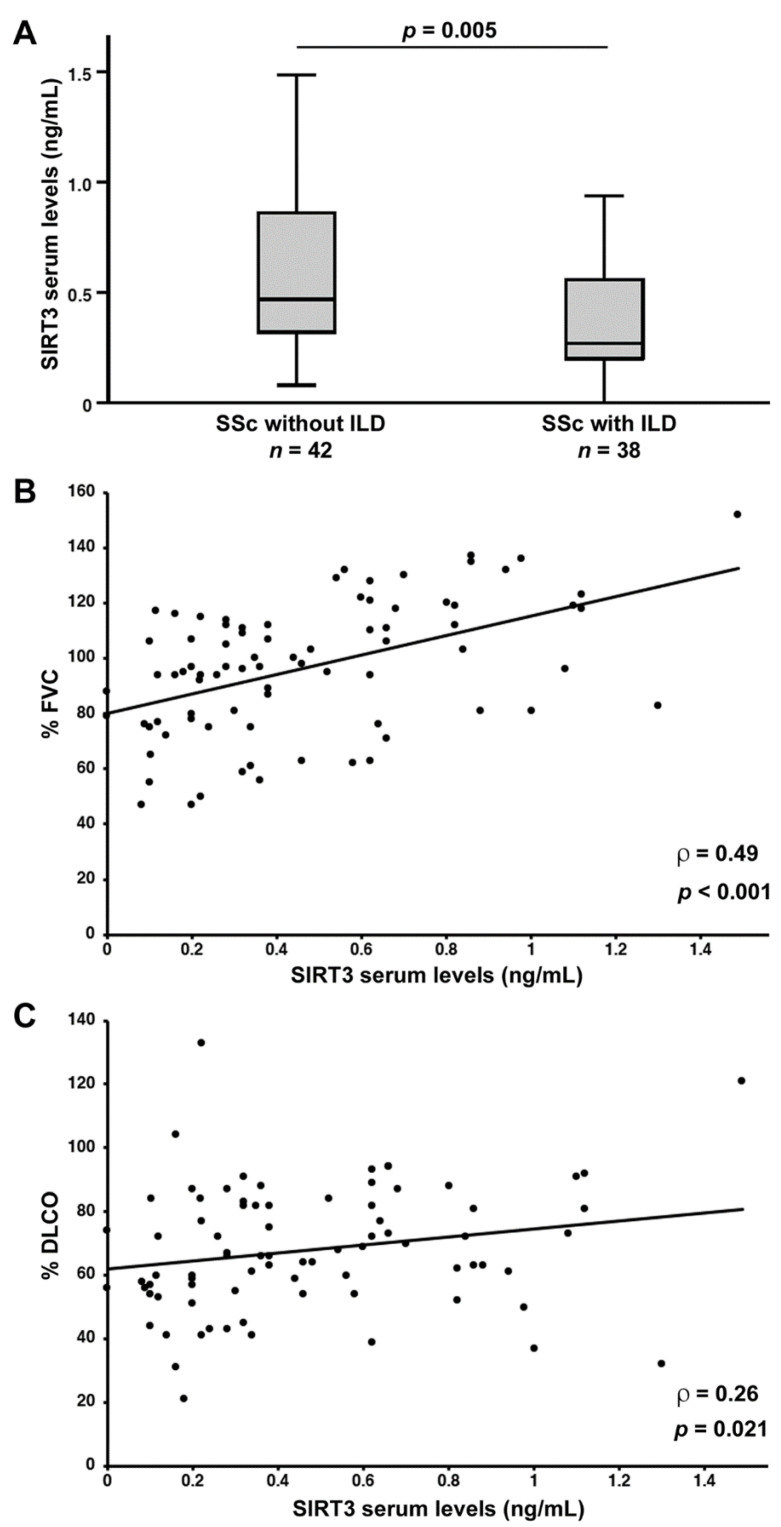
(**A**) Decreased serum levels of sirtuin 3 (SIRT3) in systemic sclerosis (SSc) patients with features of interstitial lung disease (ILD) on high-resolution computed tomography scan of the chest. Data are presented as box plots. Each box denotes the 25th to 75th percentiles. Lines outside the boxes are the 10th and 90th percentiles. Lines inside the boxes denote the median, circles denote the outliers, and asterisks denote the extreme values. (**B**) Correlation of serum SIRT3 levels with pulmonary function measured as percentage forced vital capacity (%FVC) in patients with SSc. (**C**) Correlation of serum SIRT3 levels with percentage diffusing capacity of the lungs for carbon monoxide (%DLCO) in patients with SSc. Data are displayed as scatterplot, where each dot represents a patient. Correlation coefficient (ρ) and *p* values are indicated.

As far as lung fibrosis is concerned, serum SIRT3 levels were found to be decreased in SSc patients diagnosed with ILD on HRCT scans of the chest (median 0.27 ng/mL, IQR 0.20–0.57 ng/mL) compared to those without ILD (median 0.47 ng/mL, IQR 0.32–0.86 ng/mL; *p* = 0.005; Figure 5A). Of note, in SSc patients, SIRT3 levels positively correlated with both %FVC (ρ = 0.49, *p* < 0.001; Figure 5B) and %DLCO (ρ = 0.26, *p* = 0.021; Figure 5C).

### 3.6. Association of Serum SIRT3 with the Severity of Peripheral Microvascular Damage

**Figure 6:**
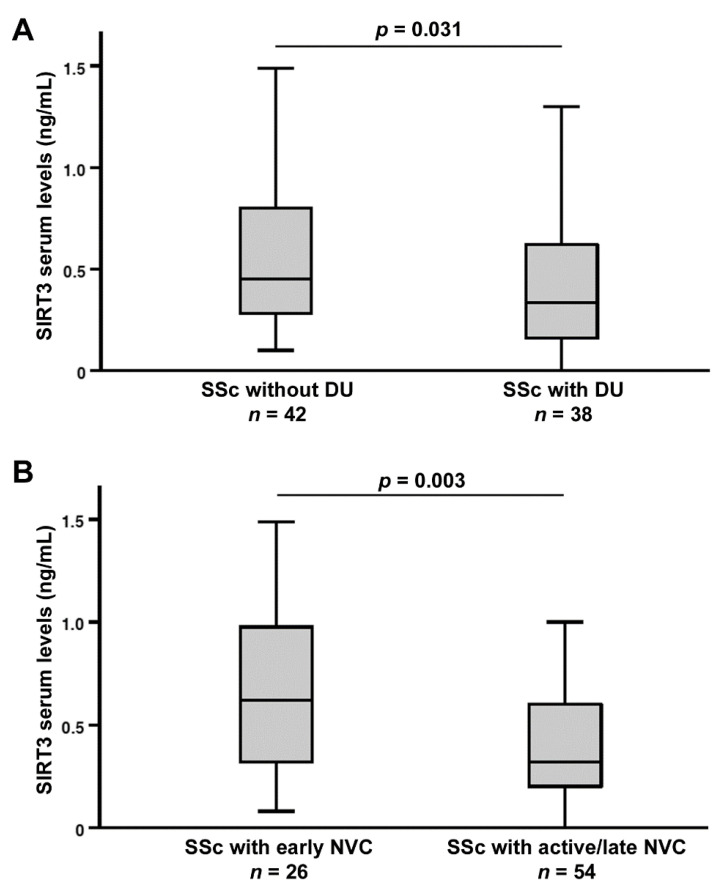
Association of serum sirtuin 3 (SIRT3) levels with the severity of peripheral microvascular damage in systemic sclerosis (SSc) patients. (**A**) Decreased SIRT3 levels in SSc patients with ischemic digital ulcers (DUs). (**B**) SIRT3 levels in SSc patients stratified according to “early” and “active/late” nailfold videocapillaroscopy (NVC) patterns. Data are presented as box plots. Each box denotes the 25th to 75th percentiles. Lines outside the boxes are the 10th and 90th percentiles. Lines inside the boxes denote the median, circles denote the outliers, and asterisks denote the extreme values; *p* values are indicated.

As far as peripheral microvascular involvement is concerned, circulating SIRT3 levels were found to be different between SSc patients with ischemic DUs (median 0.33 ng/mL, IQR 0.15–0.62 ng/mL) and those without DUs (median 0.45 ng/mL, IQR 0.28–0.80 ng/mL; *p* = 0.031; Figure 6A), as well as between SSc patients with “early” NVC (median 0.62 ng/mL, IQR 0.32–1.00 ng/mL) and those with “active/late” NVC (median 0.32 ng/mL, IQR 0.20–0.61 ng/mL; *p* = 0.003; Figure 6B).

No significant difference in SIRT3 levels was detected between SSc patients with PAH (median 0.74 ng/mL, IQR 0.20–0.86 ng/mL) and those without PAH (median 0.35 ng/mL, IQR 0.22–0.62 ng/mL; *p* > 0.05).

### 3.7. Correlation between Serum Levels of SIRT1 and SIRT3 and Logistic Regression Model

Serum levels of SIRT1 and SIRT3 were directly correlated with one another both in healthy controls (ρ = 0.27, *p* = 0.024) and in SSc patients (ρ = 0.31, *p* = 0.006).

Since, when comparing SSc subgroup medians, we found significant differences in serum levels of both SIRT1 and SIRT3 according to the cutaneous subset, the presence of ILD, and the severity of the NVC pattern, we finally carried out multiple logistic regression analysis combining serum SIRT1 and SIRT3 as independent variables, and one of the three abovementioned disease phenotypes as a single dependent variable each time. The results of logistic regression analysis are shown in Table 2.

## 4. Discussion

In this study, we provide the first evidence that circulating levels of both SIRT1 and SIRT3 are decreased in SSc patients. In particular, circulating SIRT1 and SIRT3 were found to be decreased in patients with dcSSc in respect to patients with lcSSc, and showed an association with the extent of skin involvement (i.e., mRSS), the presence of lung fibrosis on HRCT scan of the chest, and worse pulmonary function. In addition, the decrease in both serum SIRT1 and SIRT3 correlated with the severity of nailfold capillary abnormalities, with SIRT3 levels also being related to the presence of ischemic DUs. In SSc patients, we also found that serum levels of these two proteins had a direct correlation with one another. Of note, logistic regression analysis further highlighted that, of the two SIRTs, serum SIRT3 may better reflect disease activity and severity. Taken together, our findings add to those of previous experimental studies [7,13,19,20,21,22,23,24], strengthening the notion that these two SIRTs may have an important role in the development and progression of skin and pulmonary fibrosis and peripheral microvascular damage in SSc patients.

SIRTs are a family of deacetylases that, by enzymatically targeting several intracellular proteins, regulate a variety of biological processes, such as inflammation, metabolism, redox homeostasis, cell proliferation, and senescence [11,12]. Of particular interest for SSc, SIRT1 and SIRT3 have recently emerged as remarkable players in different fibrotic disorders through their ability to regulate pathways involved in fibroblast activation and tissue fibrogenesis [13]. Indeed, several reports underlie a link between decreasing SIRT1 and SIRT3 expression, and the development of fibrosis, together with a protective, antifibrotic role of SIRT restoration [13,14,15,16,17,18]. Moreover, both circulating SIRT1 and SIRT3 have been recently proposed as disease biomarkers in different pathological conditions, including those affecting the lungs [31,32,33,34,35,36,37,38].

Here, we demonstrated a decrease in circulating SIRT1 and SIRT3 levels in SSc patients with ILD respect to those without ILD, and, most importantly, a correlation of these SIRTs with %FVC, a parameter used for assessing the severity of lung restriction. Of note, among the different pulmonary function tests that have been proposed to study SSc-related ILD, only %FVC has been validated as an effective outcome measure in clinical trials [39]. Our results are in line with previous studies reporting that both SIRT1 and SIRT3 are important regulators of lung fibrosis, as they are able to attenuate TGFβ-mediated profibrotic responses both in vitro and in vivo [13,40]. The association between lower SIRT3 levels and more severe pulmonary function impairment was further strengthened by the evidence that circulating SIRT3 levels positively correlated also with %DLCO, a noninvasive test of pulmonary function that measures the ability of the lungs to exchange gas into the bloodstream. Strikingly, SIRT3 knock-out mice have been reported to present an exaggerated profibrotic response to bleomycin [24,41,42], whereas transgenic mice with whole body SIRT3 overexpression were found to be protected from bleomycin-induced lung fibrosis [41]. Finally, the recent evidence that lower SIRT3 levels may be associated with a worst prognosis in patients with COVID-19, a disease that appears to share several pathological mechanisms with SSc, further supports the potential use of serum SIRT3 as an indicator of SSc-related pulmonary disease severity [38,43,44].

Besides SIRT1 and SIRT3, SIRT7 has additionally been recently proposed as a novel regulator of lung fibrosis, and directly implicated in SSc-ILD. Indeed, a significant decline in SIRT7 expression was detected in both pulmonary fibroblasts from patients with SSc-ILD, and in lung tissues of bleomycin-challenged mice [22]. Moreover, SIRT7 silencing was also able to promote the expression of collagen and α-smooth muscle actin, whereas its overexpression significantly downregulated these profibrotic molecules by attenuating the TGFβ-induced fibrotic response in human lung fibroblasts. Combined together, the abovementioned findings suggest that SIRT7 is also protective against SSc-related lung fibrosis [22]. In view of our present findings revealing an association of circulating SIRT1 and SIRT3 reduction with the severity of SSc-ILD, we believe that whether circulating SIRT7 may show a similar trend is worth investigating in future studies.

In addition to pulmonary fibrosis, SIRT1 and SIRT3 deregulation appears to also contribute to skin fibrosis [13,19,20,21,23]. Indeed, we found that besides being lower in dcSSc patients, who present a higher extent of skin fibrosis compared to lcSSc patients, serum levels of both SIRTs also negatively correlated with mRSS, a semiquantitative method to measure skin involvement that is essential for the diagnosis and prognosis of SSc [45]. These results agree with previous studies reporting both lower SIRT1 and SIRT3 levels in the skin and explanted dermal fibroblasts from SSc patients, as well as a negative correlation between mRSS and SIRT1 mRNA levels in SSc skin biopsies [19]. Of note, another study found no correlation between SIRT3 protein expression in SSc skin and mRSS [21], a discrepancy that might be explained by the different methods and biological samples used to evaluate the levels of this protein.

There is also evidence that SIRT1 and SIRT3 play a role in regulating angiogenesis, whose profound impairment in SSc contributes to the development of ischemic peripheral microvascular disease, including DUs and NVC abnormalities [46,47,48,49,50,51,52]. In particular, SIRT1-deficient endothelial cells were found to display significantly reduced sprouting and branching capabilities, which was followed by impaired ischemia-induced neovascularization [53], whereas SIRT1 activation by resveratrol resulted in an upregulation of proangiogenic vascular endothelial growth factor in the rat myocardial infarction model [54]. Moreover, SIRT1 has been found to promote angiogenesis through deacetylation and consequent inactivation of p53 in the heart [55,56]. Similarly, SIRT3 deficiency was shown to reduce endothelial cell proliferation, capillary-like tube formation, and migration through an impairment of the glycolytic function [49,57,58], whereas SIRT3 knock-out mice manifested reduced capillary density in the heart [59]. In such a context, the association of serum SIRT1 and SIRT3 levels with the severity of SSc-related peripheral microvascular involvement is of major importance. Indeed, we found that SIRT3 levels were significantly lower in SSc patients with DUs compared to those without these ischemic complications. Furthermore, the lowest values of both circulating SIRT1 and SIRT3 were detected in SSc patients displaying the “active/late” NVC patterns, which are characterized by severe morphological changes of microvessels, and progressive capillary loss, with formation of avascular areas. All together, these results suggest that SIRT loss may actively contribute to the derangement of the peripheral microcirculation in SSc patients.

Different mechanisms, such as protein–protein interactions, NAD levels, and posttranslational modifications, may be responsible for a downregulation of SIRT expression [60]. In this context, it would be of interest to investigate whether miR-34a, a miRNA that is known to act as a translational repressor of SIRT mRNA, and to be upregulated in SSc patients [61], might be responsible for a decrease in SIRT1 and SIRT3 levels in the circulation of SSc patients.

## 5. Conclusions

In conclusion, we demonstrate that lower circulating levels of both SIRT1 and SIRT3 are associated with the extent of skin involvement, and the impairment of pulmonary function and peripheral microcirculation in SSc, with SIRT3 levels better reflecting disease activity and severity. These findings, together with those of ROC curve analysis, suggest that rather than having a diagnostic utility, these deacetylases may represent novel potential biomarkers of increased risk for a more severe, life-threatening disease course. However, further prospective studies on larger cohorts of SSc patients are necessary to examine whether, over time, changes in circulating SIRT1 and SIRT3 levels may correlate with disease progression in these patients. Whether the levels of these two molecules may also correlate with other clinical features, such as kidney function, remains to be determined. Moreover, additional in vitro and in vivo studies on preclinical animal models will help to further investigate the role of SIRT1 and SIRT3 in the pathophysiology of SSc, and to unravel if the modulation of their expression and activity might provide new targeted therapeutic approaches for the treatment of this devastating disease.

## Figures and Tables

**Table 1:** Demographic, clinical, and serological characteristics of SSc patients.

Characteristics	SSc Patients (*n* = 80)
Mean ± SD age, years	58.3 ± 13.6
Sex Male Female	6 (7.5) 74 (92.5)
Disease subset lcSSc dcSSc	47 (58.7) 33 (41.3)
Autoantibody positivity ANAs ACAs ATAs	75 (93.7) 39 (48.7) 30 (37.5)
Digital ulcers	38 (47.5)
Capillaroscopy pattern “Early” “Active” “Late”	26 (32.5) 33 (41.2) 21 (26.2)
Mean ± SD mRSS	10.7 ± 9.0
Mean ± SD %FVC	96.6 ± 23.8
Mean ± SD %DLCO	67.9 ± 19.8
ILD	38 (47.5)
PAH	12 (15.0)

Except where indicated otherwise, values are *n* (%) of subjects. ACAs, anticentromere antibodies; ANAs, antinuclear antibodies; ATAs, antitopoisomerase I antibodies; dcSSc, diffuse cutaneous SSc; DLCO, diffusing capacity of the lungs for carbon monoxide; FVC, forced vital capacity; ILD, interstitial lung disease; lcSSc, limited cutaneous SSc; mRSS, modified Rodnan skin thickness score; PAH, pulmonary arterial hypertension; SSc, systemic sclerosis.

**Table 2:** Logistic regression analysis model combining serum SIRT1 and SIRT3 levels.

		dcSSc	ILD	“Active/Late” NVC
SIRT1	OR (95% CI)	0.69 (0.39–1.24)	0.73 (0.43–1.23)	0.65 (0.39–1.08)
	*p*	0.22	0.23	0.09
SIRT3	OR (95% CI)	0.08 (0.01–0.53)	0.13 (0.02–0.75)	0.12 (0.02–0.64)
	*p*	0.009	0.022	0.013

CI, confidence interval; dcSSc, diffuse cutaneous systemic sclerosis; ILD, interstitial lung disease; NVC, nailfold videocapillaroscopy; OR, odds ratio; SIRT, sirtuin.

## Data Availability

All relevant data are included within the manuscript.
